# Endothelial dysfunction and preeclampsia: role of oxidative stress

**DOI:** 10.3389/fphys.2014.00372

**Published:** 2014-10-10

**Authors:** Lissette C. Sánchez-Aranguren, Carlos E. Prada, Carlos E. Riaño-Medina, Marcos Lopez

**Affiliations:** ^1^Translational Biomedical Research Group, Biotechnology, Innovation and Technology Development, Cardiovascular Foundation of ColombiaFloridablanca, Colombia; ^2^Graduate Program in Biomedical Sciences, Faculty of Health, Universidad del ValleCali, Colombia; ^3^Center for Genomic Medicine and Metabolism, Instituto del Corazón de Floridablanca, Cardiovascular Foundation of ColombiaFloridablanca, Colombia; ^4^Division of Human Genetics, Cincinnati Children's Hospital Medical Center, University of Cincinnati College of MedicineCincinnati, OH, USA; ^5^Maternal-Fetal Medicine Program, Cardiovascular Foundation of ColombiaFloridablanca, Colombia

**Keywords:** preeclampsia, endothelial dysfunction, oxidative stress, reactive oxygen species, superoxide

## Abstract

Preeclampsia (PE) is an often fatal pathology characterized by hypertension and proteinuria at the 20th week of gestation that affects 5–10% of the pregnancies. The problem is particularly important in developing countries in where the incidence of hypertensive disorders of pregnancy is higher and maternal mortality rates are 20 times higher than those reported in developed countries. Risk factors for the development of PE include obesity, insulin resistance and hyperlipidemia that stimulate inflammatory cytokine release and oxidative stress leading to endothelial dysfunction (ED). However, how all these clinical manifestations concur to develop PE is still not very well understood. The related poor trophoblast invasion and uteroplacental artery remodeling described in PE, increases reactive oxygen species (ROS), hypoxia and ED. Here we aim to review current literature from research showing the interplay between oxidative stress, ED and PE to the outcomes of current clinical trials aiming to prevent PE with antioxidant supplementation.

## Preeclampsia: a disorder of global impact

Preeclampsia (PE) is a dangerous complication of pregnancy clinically detected on the second half of gestation. PE is a disorder that affects 5–10% of pregnancies and is characterized by hypertension (Walker, 2000; North et al., 2011) and proteinuria at the 20th week of gestation (Redman and Sargent, 2005). According to the World Health Organization (WHO), 20% of the 15 million preterm births reported each year are related to PE (Kinney et al., 2012; Liu et al., 2012). This situation is particularly important in developing countries where the incidence of hypertensive disorders of pregnancy is higher and maternal mortality rates and preterm births are 20 times higher than those reported in developed countries (Walker, 2000; Lain and Roberts, 2002).

Classical conditions and risk factors such as nulliparity, maternal age, insulin resistance, deficient nutrients intake such as calcium or antioxidant vitamins, subclinical infections, metabolic syndrome, genetic predisposition or immune factors participate independently or in association to increase the risk to develop PE (Eskenazi et al., 1991; López-Jaramillo et al., 2005). Nowadays, even in low and middle income countries, unhealthy lifestyles along with the accessibility to low-cost high caloric and fat containing meals contribute to metabolic dysregulation and maternal obesity (Monteiro et al., 2004). While in developed countries changes in lifestyles and diets were incorporated gradually for decades, in low and middle income countries, these changes were drastic and fast (≤10 years). Beyond the classical conditions for the development of PE, now, more specific risk factors such as anti-angiogenic factors release, maternal malnutrition and epigenetics, are hot topics of research.

Poor maternal health and nutrition predispose women to pregnancy difficulties like PE and gestational diabetes (Roberts et al., 2003). Maternal health and nutrition is very important because they have a direct impact on the placental environment, fetal development and child's overall health later in life (Barker and Osmond, 1986). Utero fetal programming (Barker hypothesis) due to poor health and malnutrition during pregnancy may affect organ development and growth. Current evidence have established that disorders like hypertension, metabolic syndrome, type 2 diabetes, vascular disease and predisposition to develop PE are a result of fetal reprogramming due to poor health and malnutrition during pregnancy (Godfrey et al., 1996; Desai and Hales, 1997; López-Jaramillo and López-López, 2010). Certainly, the manifestation of these disorders in later stages in life is triggered by sedentary lifestyles and environmental risk factors. This is particularly important in order to understand the etiology of PE in undeveloped countries in where changes in lifestyles and environmental factors could be rapid and drastic.

Interestingly, the “diversity” of conditions that may trigger PE, are population, race and country dependent (Sibai et al., 1997; López-Jaramillo et al., 2005). While in developed countries the prevalent factors that accompany the development of PE are associated with obesity, insulin resistance and hyperlipidemia, in developing countries ethnicity, poor nutritional habits, and subclinical infections along with other socioeconomical factors are usually associated with the development of the disease. Even the outcomes of clinical trials to prevent PE are region and population dependent. This is very important because it demonstrates that since PE genesis may have distinct regional or population specific causes and risk factors, the strategies to prevent PE should be specific as well (López-Jaramillo et al., 2005; Liu et al., 2012). However, most of these factors are associated with impaired endothelial function and abnormal placentation that are key events in the development of PE. Despite all this, the etiology and pathophysiological mechanisms of PE are still unknown.

## Pathophysiology of preeclampsia: endothelial dysfunction

Normal pregnancy course include variations in hemodynamics, like heart rate and cardiac output (Duvekot et al., 1993) in which placenta allows the exchange of nutrients and waste disposal between mother and fetus (Myatt, 2010). This maternal-fetal interface is developed during the first trimester of gestation. Then, extravillous throphoblasts from placenta conquer the maternal decidua. During this stage, the maternal spiral arteries from the decidua go through a process of remodeling in where they are upgraded from low-capacity high-resistance into high-capacity low-resistance vessels. This is also accompanied by the substitution of arterial smooth muscle and elastic tissue with fibrinoid material. PE in turn is characterized by an impaired invasion of fetal trophoblasts. This causes a reduced remodeling of the maternal spiral arteries eventually leading to a decrease in blood flow to the placenta. This affects the fetus and placental oxygen and nutritional status. In order to compensate the blood flow deficiency, the mother develops hypertension to increase the blood flow, usually at the end of the second or third trimester of gestation. Interestingly, problems associated with the disorder cease after delivery, suggesting that PE is a problem that originates from the placenta (Redman, 1991).

Pregnancy physiology requires proper placental oxygenation. However, ROS, derived from these high fluxes of oxygen, are implicated and required for replication, proliferation and cell maturation, embryo development and pregnancy maintenance (Mutinati et al., 2013). Moreover, increase in oxygen concentrations results in the appearance of markers of oxidative stress (Redman and Sargent, 2005; Yang et al., 2012).

It is widely accepted that PE originate from placenta and specifically from trophoblast cells. These cells, that are placental native (Redman and Sargent, 2005), are the reunion of two subtypes: syncytiotrophoblasts, that are responsible for the formation of a primary external layer in direct contact with maternal blood, and cytotrophoblasts, that conform the inner layer, and differentiate and invade the maternal endometrial stroma (Hunkapiller and Fisher, 2008). Poor placental perfusion due to irregularities in the process of placentation and trophoblast invasion during the development of placenta, have been associated with hypertension in early stages of pregnancy (Karthikeyan and Lip, 2011). These abnormalities in the perfusion of placenta, can lead to changes in this fetal-derived organ, that can activate or repress normal functions of endothelial cells (Roberts et al., 1991).

**Figure 1:**
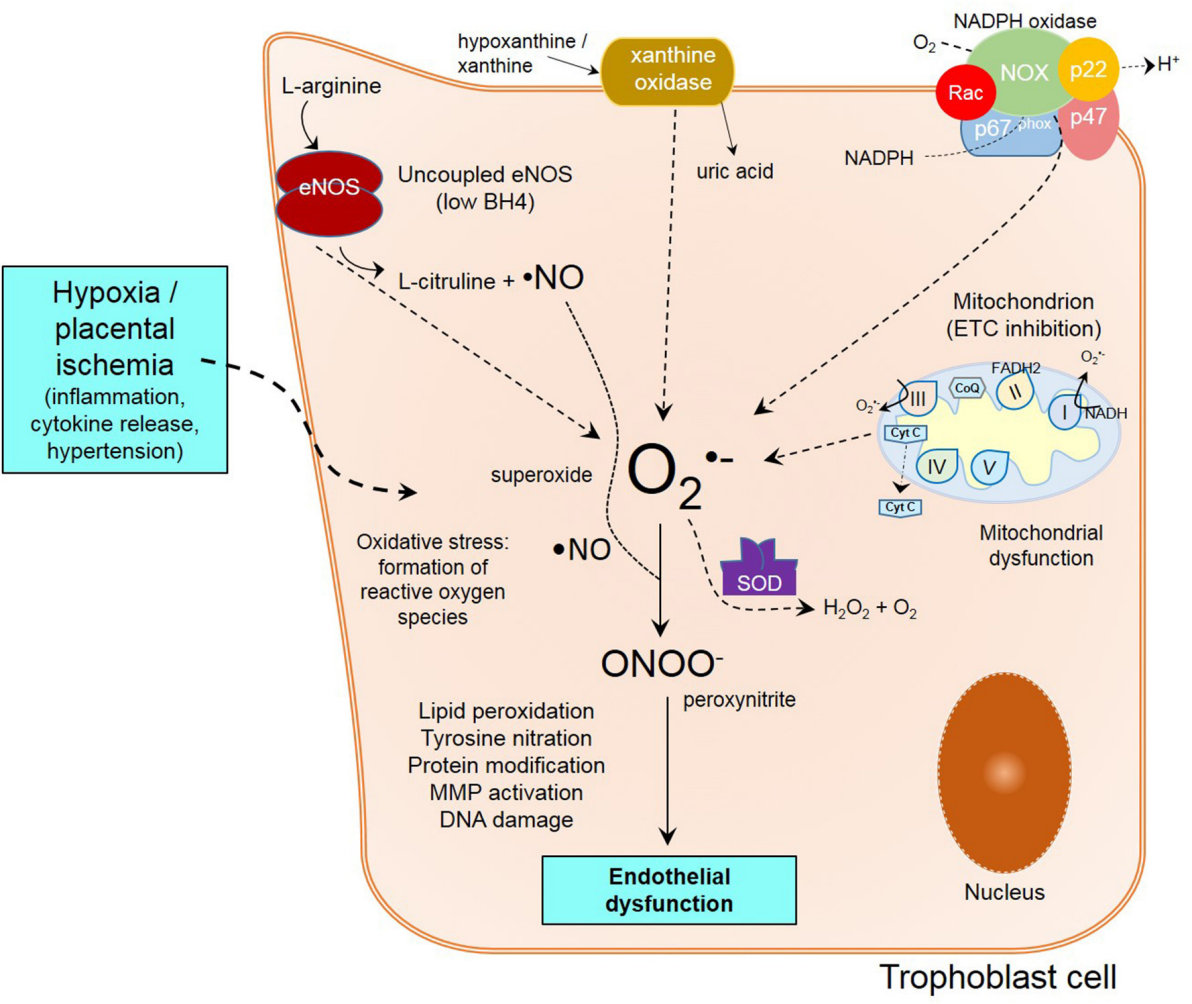
**Mechanisms of endothelial dysfunction in preeclampsia**. Poor trophoblast invasion in PE causes impaired spiral artery remodeling following by placental ischemia/reperfusion and inflammation. Within the trophoblast cell, oxidative stress from unbalanced free radical formation is formed from different sources like XO, eNOS uncoupling, NADPH oxidase, and mitochondria. Ultimately, the reunion of all these events lead to peroxynitrite formation, lipid peroxidation, protein modification, MMP activation and DNA damage, contributing to endothelial dysfunction.

In normal conditions, remodeling of maternal spiral arteries is necessary to access maternal blood supply (Lyall et al., 2013). Nevertheless, impair remodeling of arteries and poor development of placenta caused by shallow trophoblastic invasion (Verlohren et al., 2010), are associated with the establishment of preeclampsia and generalized maternal endothelial and vascular dysfunction (Redman and Sargent, 2003; Myatt and Webster, 2009; Saito and Nakashima, 2014). The reduced placental perfusion seen under this circumstances, creates changes in the placental environment, in where ROS, and the activation of endothelial cells through different mechanisms, results in ED. Due of defective trophoblast invasion, intermittency of arterial blood flow occurs, resulting in periods of ischemia/reperfusion, creating a hypoxic environment which favors oxidative stress, consequent oxidative damage and inflammation (Myatt and Webster, 2009) (Figure 1).

**Figure 2:**
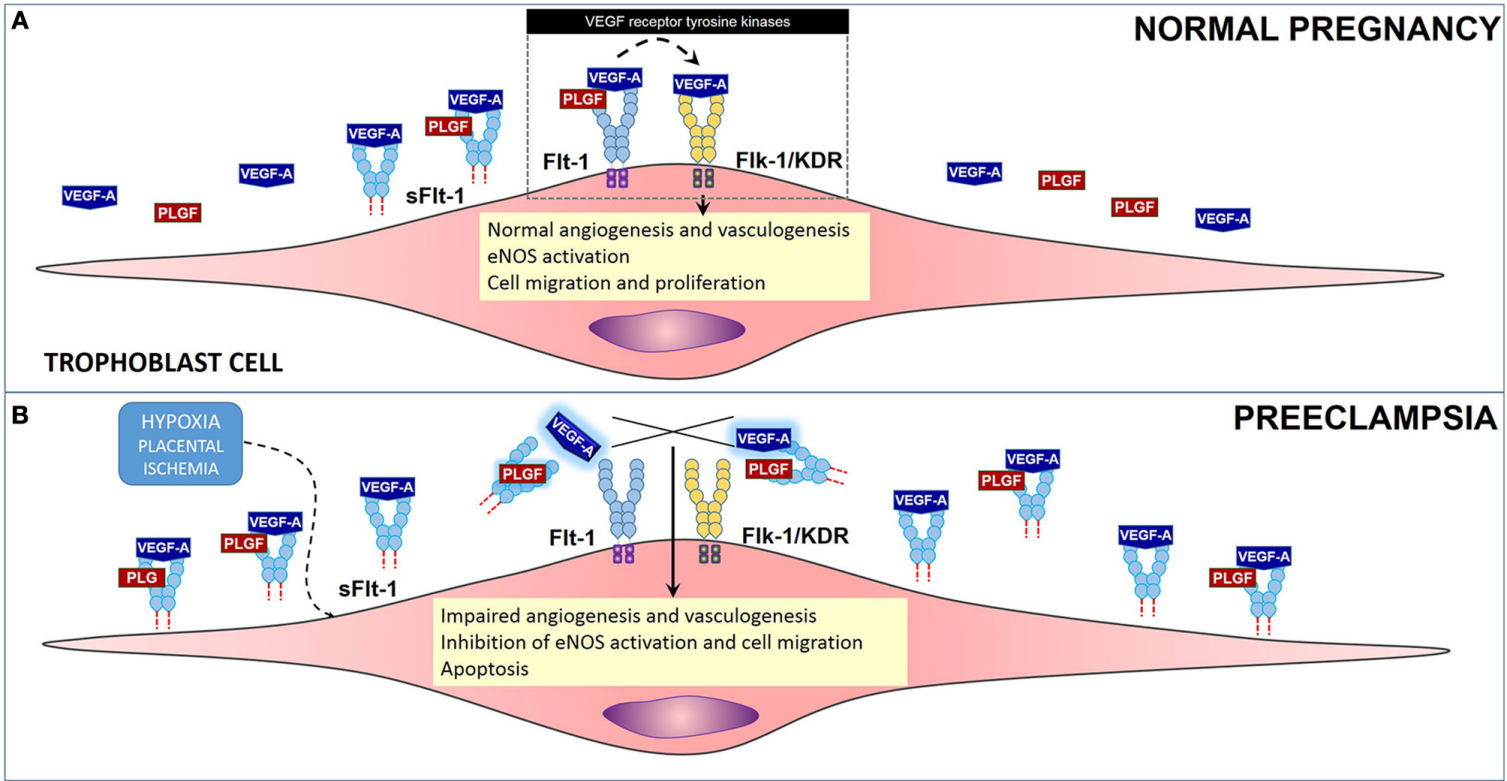
**Role of anti-angiogenic factor sFlt-1 in preeclampsia. (A)** Flt-1 (light blue) and Flk-1 (yellow), are VEGF receptor tyrosine kinases that regulate the process of angiogenesis and vasculogenesis, among other events in cells in PE. Soluble form of VEGF receptor 1, sFlt-1 under normal conditions regulates VEGF levels, angiogenesis and vasculogenesis. **(B)** Under hypoxic conditions, Flt-1 is cleaved producing sFlt-1 in high concentrations. sFlt-1 then competes with Flt-1 for binding of VEGF-A and PlGF causing an impairment in the angiogenesis process by decreasing the bioavailability of VEGF-A and PlGF to Flt-1 and Flk-1.

One of the mechanisms of ED involves release of the sFlt-1 (soluble fms-like tyrosine kinase or sVEGFR1), which is a circulating anti-angiogenic protein and an endogenous inhibitor of vascular endothelial growth factor (VEGF), that works by enhancing the ED already established by oxidative stress, ROS and damage (Sato et al., 2000; Luttun et al., 2002; Maynard et al., 2003; López-Novoa, 2007; Widmer et al., 2007; Zhou et al., 2011; Murphy et al., 2013) (Figure 2). VEGF is key in the process of growth of new blood vessels and in the overall maintenance and endothelial cell health. Levels of sFlt-1 are known to be increased in PE, and this increase precedes disorder manifestation. High levels of this VEGF inhibitor, causes a disruption on VEGF by sticking to endothelial cell Flt-1 receptor, found on membrane. sFlt-1 is a truncated form of the Flt1 receptor. sFlt-1 when secreted, antagonizes VEGF and placental growth factor (PlGF), enhancing ED. Several studies have shown that VEGF and PlGF are downregulated in PE by sFlt-1 (Sato et al., 2000; Luttun et al., 2002; Maynard et al., 2003; López-Novoa, 2007; Widmer et al., 2007; Murphy et al., 2013).

Studies in cells (HUVECs), supplemented with serum from preeclamptic and normotensive women, have shown that in ones treated with serum from the PE group, due to increased levels of sFlt-1, tube-like structure formation is impaired. However, ones treated with normotensive serum formed normal regular tube-like structures. In contrast, the effect of sFlt-1 inhibition of tube-like structures was rescued by exogenous VEGF and PlGF supplementation to the preeclamptic serum. Additionally, the same group performed an *in vivo* experiment with pregnant rats that were injected with sFlt-1. The results from these experiment correlate with the findings shown in cells in where the treated group developed PE-like symptoms, significant hypertension and heavy albuminuria and downregulation of PlGF (Maynard et al., 2003).

Despite all these recent findings the genesis of ED in PE still an enigma (Nagamatsu et al., 2004; Widmer et al., 2007). However, recently, another interesting study explored the effects of hypoxia on the regulation of VEGF, PlGF, and sFlt-1, in isolated cytotrophoblasts, HUVECs and villous fibroblasts. Results demonstrated that hypoxic conditions induced expression of sFlt-1 in cytotrophoblasts but not in HUVECs and villous fibroblasts. These demonstrate that under hypoxic conditions cytotrophoblasts are responsible for orchestrating the downregulation of VEGF, PlGF, and the upregulation of sFlt-1. Certainly, these results provide clarification on the discussion on the perturbations of the remodeling of maternal spiral arteries that occur in PE. Nevertheless, how all these events occur and concur to induce the following events: (1) a defective trophoblast invasion that may result in an intermittency of arterial blood flow, (2) periods of ischemia/reperfusion, (3) the creation of a hypoxic environment which favors oxidative stress, (4) consequent oxidative damage, (5) an inflammatory response, and finally (6) the release of sFlt-1 and the downregulation of VEFG and PlGF; are questions still open for further research.

## Oxidative stress and preeclampsia

ROS, like nitric oxide (^·^NO), superoxide (O^·−^_2_), hydrogen peroxide (H_2_O_2_), hydroxyl radical (^·^OH), and peroxynitrite (ONOO^−^), are signaling molecules that regulate many functions in human physiology (Kalyanaraman, 2013). ROS signaling is directly controlled by antioxidant host defenses that scavenge the actions of these species. During normal gestation, ROS generation are known to be increased and necessary for proper physiology (Yang et al., 2012). However, a whole different story occurs when the balance between our antioxidant host defenses and the pro-oxidant species is broken, like in PE. The process in where the relative pro-oxidant species called ROS are much higher than the antioxidant army defenses, is called oxidative stress (Myatt and Cui, 2004; Lappas et al., 2010; Matsubara et al., 2010; Kalyanaraman, 2013).

As in any vascular disease, PE is characterized by a resulting inflammatory response after ischemia and reperfusion (Redman, 1991; Webster et al., 2008; Poston et al., 2011). In PE, placental reperfusion injury converges into a damaging inflammatory response that is responsible for inflammation and oxidative damage orchestrated by oxidative stress. Immediately after placental reperfusion injury, reestablished blood flow releases cytokines and other inflammatory factors like tumor necrosis factor-alpha (TNF-α), interleukin (IL)-6, and IL-10, C-reactive protein (CRP), and damaging levels of ROS like superoxide, in response to these events. Increased ROS may eventually trigger a redox signaling process to induce cell apoptosis. Scientific evidence suggest that reduced perfusion due to aberrant placentation and shallow trophoblast invasion, triggers a condition of placental oxidative stress (Yiyenoǧlu et al., 2013) leading to intravascular inflammatory response and endothelial dysfunction. Taken together, these situations are probably involved in the etiopathogenesis of PE.

Oxidative stress causes post-translational covalent modification of protein (Roberts et al., 2009; Myatt, 2010) and DNA, and damage in protein and lipid structure and function (Jones et al., 2013). The existence of ROS during normal gestation is a fact (Yang et al., 2012), indicating that an impairment of the natural antioxidant defense mechanism is probably implied in PE (Karacay et al., 2010). Consistent with these facts, elevated concentrations of thioredoxin-1, a redox-sensitive protein that regulates biological functions, is associated to high oxidative stress conditions in pregnant women (Nakatsukasa et al., 2013). Other oxidative markers such as malondialdehyde, a marker of lipid peroxidation and prostaglandin F in serum from women with 10—14 gestation weeks, were found to be increased in preeclamptic women. This correlates with the gradual oxidative damage of the placenta, even before the onset of clinical symptoms (Genc et al., 2011).

## Role of ^·^NO and nitric oxide synthase (NOS)

Not all free radicals cause disturbances in the organism (Kalyanaraman, 2013) and ^·^NO is an example (Palmer et al., 1988; Moncada et al., 1991; Moncada and Higgs, 1993). ^·^NO is a potent vasodilator, that causes relaxation of smooth muscle (Seligman et al., 1994). It mediates endothelial function by regulating vascular tone, platelet aggregation, leukocyte adhesion and smooth muscle cells development (Qian and Fulton, 2013). It is synthetized by the NOS family of enzymes, which consist in three isoforms: nNOS or neuronal isoform, iNOS, the inducible and eNOS endothelial NOS (Qian and Fulton, 2013) from the reduction of L-arginine to L-citruline (Palmer et al., 1988; Moncada et al., 1991; Moncada and Higgs, 1993). In placenta, eNOS expression is associated with cytotrophoblast to syncytotrophoblast differentiation (Eis et al., 1995).

The role of ^·^NO in PE has not been established as in other vascular systems. Results obtained from studies detecting ^·^NO levels in maternal plasma, serum or urine from women with PE have been controversial and variable. As resumed in Table 1, results coming from different groups have reported different ^·^NO levels (as NO^−^_3_/NO^−^_2_ usually via Griess reaction or chemiluminescence) from different sources like plasma, serum or urine. As shown in Table 1, at this point there is no consensus on the expected ^·^NO levels in PE. The detected levels vary from study to study and tissue/fluid of determination. In the sample of studies we assessed, usually most sampling was performed either in serum or plasma and detection method of choice was the Greiss method with the exception of three studies which used a Siever's Nitric Oxide Analyzer or GC-MS. As a general trend, most studies showed that ^·^NO levels were significantly lower in preeclamptic than normotensive women (Seligman et al., 1994; Baker et al., 1995; Davidge et al., 1996; Nobunaga et al., 1996; Pathak et al., 1999; Nishikawa et al., 2000; Shaamash et al., 2000; Vural, 2002) with a few exceptions in where levels were not significantly different (Lyall et al., 1995; Conrad et al., 1999; Diejomaoh et al., 2004). Studies from Ecuador, Brazil and China, have shown that ^·^NO levels were significantly lower in preeclamptic than normotensive women (Teran et al., 2006; Mao et al., 2010; Sandrim et al., 2010). This latter studies were performed using more sensitive and selective techniques like a Siever's Nitric Oxide Analyzer or GC-MS. However, most studies have correlated their results with blood pressure determinations.

**Table 1:** **^·^NO concentration results from different studies in PE**.

**Reference**	**Method**	**Tissue/Fluid**	**^·^NO levels**					**Country**
			**Preeclamptic**	***N***	**Normotensive**	***N***	***p***	
Seligman et al., 1994	Griess	Serum	4.65 ± 0.85 μmol/L	26	3.46 ± 1.43 μmol/L	26	0.02	US
Baker et al., 1995	Griess	B88 cells	97.3 ± 9.6 nmol/mg	10	71.9 ± 4.3 nmol/mg	10	<0.05	US
Lyall et al., 1995	Griess	Serum	29.5 ± 1.06 μmol/L	32	29.8 ± 1.07 μmol/L	36	NS	UK
Davidge et al., 1996	Griess	Plasma Urine	32.7 ± 3.1 μmol/L	14	25.8 ± 2.4 μmol/L	20	NS	US
			0.37 ± 0.06 μmol	14	0.69 ± 0.11 μmol	20	<0.05	
			NO^−^_2_/mg creatinine		NO^−^_2_/mg creatinine			
Nobunaga et al., 1996	Griess	Plasma	45.6 ± 2.3 μmol/L	23	30.3 ± 1.0 μmol/L	323	<0.01	Japan
Pathak et al., 1999	Griess	Serum	11.82 ± 1.16 μmol/L	50	5.08 ± 0.47 μmol/L	50	<0.01	India
Choi et al., 2002	Griess	Serum	43.1 ± 12.7 μM	52	249.7 ± 51.3 μM	80	<0.05	Korea
Aydin et al., 2004	Griess mod.	Plasma	48.11 ± 3.77 μmol/L	35	63.14 ± 7.08 μmol/L	34	<0.001	Turkey
Diejomaoh et al., 2004	Griess mod.	Serum	19.189 ± 16.805 μmol/L	34	19.157 ± 13.407 μmol/L	39	NS	Kuwait
Sandrim et al., 2010	Sievers NOA	Plasma	102 ± 7.1 nmol/L	47	214.8 ± 26.1 nmol/L	47	<0.05	Brazil
Ehsanipoor et al., 2013	Griess	Plasma	36.5 μM	12	58.1 μM	13	<0.0001	US
Conrad et al., 1999	Griess	Plasma	35 ± 2 μM	15	34 ± 2 μM	22	NS	US
Nishikawa et al., 2000	Griess	Serum	43.23 ± 3.55 μM	17	23.63 ± 1.87 μM	16	NS	Japan
Shaamash et al., 2000	Griess	Serum	28.3 ± 2.6 μmol/L	31	20.5 ± 6.7 μmol/L	32	<0.001	Egypt
Vural, 2002	Griess	Plasma	88.83 ± 5.67 μmol/L	19	62.63 ± 9.52 μmol/L	20	<0.001	Turkey
Teran et al., 2006	Sievers NOA	Plasma	15.8 ±1.1 μM	30	23.4 ± 1.9 μM	60	<0.01	Ecuador
Mao et al., 2010	GC-MS	Plasma	23.42 ± 2.86 μmol/L	60	28.83 ± 2.44 μmol/L	30	<0.01	China

Other newer studies have followed the same trend and have tried to find further explanations by correlating their results with cell or tissue studies from placenta or umbilical cord. Recently, a study performed on a Mexican cohort, have shown that the plasma levels of ^·^NO, measuring the levels of NO^−^_3_/NO^−^_2_, were found to be higher in PE women than normotensive (Gonzalez-Garrido Chem et al., 2013). In comparison, a recent study performed in a Brazilian cohort measuring plasma ^·^NO using the Greiss reagent, have shown that ^·^NO levels are lower in PE women (Pimentel et al., 2013). In contrast, in the same Mexican cohort, ^·^NO levels were found to be lower in endothelial cells obtained from preeclamptic umbilical cords in comparison to normal pregnancies. Therefore, a small study performed in placenta using Electron Paramagnetic Resonance (EPR) evidenced a reduction of ^·^NO concentration in PE placentas of almost half of the concentration when compared to normal placentas. However, *in vitro*, expression of inducible NOS (iNOS) mRNA was increased in cells treated with serum from preeclamptic women (Matsubara et al., 2010). On another study, the expression (Davidge et al., 1995) as well as the activity of nitric oxide synthase was significantly increased in endothelial cells exposed to preeclamptic plasma (Baker et al., 1995). As evidenced, ^·^NO levels in PE women are still controversial and several reports have shown in different cohorts that in comparison with normotensive women, levels are either the same, higher or lower than the PE group (López-Jaramillo et al., 2008).

On the other hand, low levels of substrate L-arginine and high levels of the endogenous eNOS inhibitor asymmetric dimethylarginine (ADMA) might interfere with eNOS activity during preeclampsia. Significantly lower levels of L-arginine have been shown in preeclamptic women, while plasma levels of ADMA were no significantly different between normal and preeclamptic women in various studies (Maas et al., 2004; Kim et al., 2006; Khalil et al., 2013). However, in a Greek cohort the plasma ADMA levels in PE women were found to be higher (Savvidis et al., 2011). Again, in contrast, in a Brazilian cohort, plasma ADMA levels were found to be also higher in PE women and plasma ^·^NO levels were found to be lower in comparison with normotensive women (Sandrim et al., 2010).

Additionally, eNOS uncoupling have also been shown as a source of superoxide formation and it is related to reduced ^·^NO production (Vasquez-Vivar et al., 2003; Yzydorczyk et al., 2013) when eNOS cofactor, tetrahydrobiopterin (BH4) is low (Karbach et al., 2014) or when post-translational changes regulate eNOS function (Qian and Fulton, 2013). It has been demonstrated that various inflammation modulators like TNF-α and CRP are increased in plasma (Teran et al., 2001; Garcia et al., 2007; Sorokin et al., 2010) and placenta from PE women (Hung et al., 2008). TNF-α downregulates eNOS and mitochondrial biogenesis leading to mitochondrial dysfunction (MD) and elevated ROS (Valerio et al., 2006). Conversely, CRP indirectly downregulates BH_4_ production, leading to eNOS uncoupling and peroxynitrite formation (Singh et al., 2007; Jialal et al., 2009). However, the role of BH4, eNOS and ^·^NO production is still not very well understood in PE. BH4 promotes eNOS dimerization and activity. In a rat model of pregnancy-induced hypertension it has been demonstrated that supplementation with BH4 as sepiapterin increased ^·^NO levels, and reduced O^·−^_2_ and ONOO^−^ production (Mitchell et al., 2007). Nevertheless, in placenta, the story is different. eNOS activity and levels have been measured in placenta from PE and normotensive women by different accepted methods (Conrad and Davis, 1995; Kukor et al., 2000; Kim et al., 2006). Nonetheless, as with other determinations, the results are contradictory, from been lower or higher in some cohorts and the same in others.

## Role of superoxide (O^·−^_2_): xanthine oxidase, NADPH oxidase, and mitochondria

O^·−^_2_ is a free radical of great biological importance. It is produced by the one electron reduction of oxygen. In cells it is one of the secret weapons used by the immune system army to kill invading pathogens. The main quencher of O^**·**−^_2_ is antioxidant superoxide dismutase (SOD) that coverts it to H_2_O_2_ and water. H_2_O_2_is immediately neutralized by catalase (CAT). However, O^·−^_2_ is also produced by several pathological conditions including PE. After ischemia, reperfusion causes oxidative damage mainly by the conversion of xanthine dehydrogenase (XD) to xanthine oxidase (XO). In parallel, in ischemic tissues, hypoxanthine (HX) is formed as breakdown product of ATP metabolism. XO converts xanthine or HX to uric acid and oxygen to O^·−^_2_ and H_2_O_2._ In placenta, the situation is even more complicated because during pregnancy, iron is transferred from the mother to the fetus across the placenta. Iron is trafficked across the placenta from dietary or endogenous maternal sources to meet fetal demands (Cao et al., 2013). In contrast with enterocytes, placenta must restrict iron uptake into the syncytiotrophoblast by storing extra iron within tissue until parturition or exporting excess intracellular iron back into maternal circulation. Iron and other metals catalyze the production of a more damaging potent pro-oxidant species called hydroxyl radical (^·^OH) via the Fenton reaction (Many et al., 1996, 2000; Webster et al., 2008; Kalyanaraman, 2013).

In PE, superoxide generation by XO has been shown in placental reperfusion injury (Many et al., 2000). Since, PE is characterized by hyperuricemia, XO is presumably source of uncontrolled ROS production (Many et al., 1996) when the concentration of its oxidase form is increased. Studies have shown in cytotrophoblast cells from preeclamptic women that the activity of xanthine oxidase is increased compared to control cells (Many et al., 2000; Yildirim et al., 2004; Bainbridge et al., 2009; Mills et al., 2009).

Another source of O^·−^_2_ formation are NADPH oxidases. NADPH oxidase is a membrane-bound enzyme complex that catalyzes the one-electron reduction of oxygen to O^·−^_2_ via NADPH (Babior, 2004). It has been demonstrated that NADPH oxidase isoform NOX1 is overexpressed in syncytiotrophoblast of preeclamptic placentas (Cui et al., 2006). In an *in vitro* model, it has been shown that HUVEC cells treated with serum from preeclamptic women increased expression of NADPH oxidase subunit gp91 (phox), leading to high amounts of O^·−^_2_ production (Matsubara et al., 2010). In addition, NOX2 overexpression have been evidenced in cultured primary cultured HUVECs from normal and preeclamptic pregnancies (Choi et al., 2013). In PE, NADPH activation is triggered by angiotensin II (Ang II) signaling that leads to inflammation. Ang II stimulates NADPH oxidase through the AT1 receptor AT1-AA by causing the placenta to produce ROS, activate nuclear factor kappa B (NF-κ B) and trigger inflammation (Dechend et al., 2003). Several other reports have concur that in PE women NADPH oxidase activity is increased (Matsubara and Sato, 2001; Lee et al., 2003; Myatt and Cui, 2004; Raijmakers et al., 2004; Cui et al., 2006) and thus an important source of O^·−^_2_ formation.

Mitochondrion is a very important organelle because is responsible for the production of ATP through respiration and regulating cell metabolism. Mitochondrial activity is essential in pregnancy because it sustains the metabolic activity of the placenta throughout these period (Mandò et al., 2014). As in many other scenarios, under pathological conditions, mitochondria is another source of O^·−^_2_ formation contributing to placental damage (Torbergsen et al., 1989; Colleoni et al., 2013; Maranzana et al., 2013; Mayeur et al., 2013; Pimentel et al., 2013; Mandò et al., 2014). After reperfusion injury, re-oxygenation induces tissue and mitochondrial damage. As in other vascular diseases, mitochondrial dysfunction is also evidenced in PE (Torbergsen et al., 1989). Some sources of O^·−^_2_ formation in mitochondria under pathological conditions include complexes I and II of the mitochondrial transport chain (Myatt, 2010; Maranzana et al., 2013). Nevertheless, changes in preeclamptic placenta proteome, related to the respiratory chain and ROS generation may explain the importance of mitochondria in the development of preeclampsia (Shi et al., 2013). Thus, mitochondrial function is disturbed in hypoxic placentas (Colleoni et al., 2013). It has been shown that changes in oxygen consumption rate (OCR) measured by a Seahorse Flux Analyzer indicate mitochondrial dysfunction in trophoblasts isolated from preeclamptic placentas in comparison with normotensive ones (Maloyan et al., 2012; Muralimanoharan et al., 2012).

Beyond its role as a source of oxidative stress, mitochondria is known to be affected by exposure to suboptimal environmental conditions as in PE. Placental metabolism is maintained throughout gestation by increasing mitochondrial biogenesis and activity (Mandò et al., 2014). Mitochondrial dysfunction disrupts fundamental processes important for embryo development and, in turn, it has a direct effect on fetal and placental growth and function. Mitochondrial dysfunction has been shown to be a key factor for fetal programming in situations of placental insufficiency like PE (Mandò et al., 2014). In mouse models, it has been demonstrated that mitochondrial dysfunction disturbs fetal and placental growth and development (Wakefield et al., 2011; Mayeur et al., 2013). Conversely, in rat models, it has been demonstrated that poor gestational nutrition induces mitochondrial dysfunction that in turn is implicated partially in fetal growth restriction (Mayeur et al., 2013; Pimentel et al., 2013).

## Role of peroxynitrite (ONOO^−^)

Although O^·−^_2_ anion exert major tissue damage in placenta, its reaction with ^·^NO to produce ONOO^−^, a strong pro-oxidant agent, has gained importance in many vascular diseases like PE. ONOO^−^ is produced *in vivo* by the reaction of ^·^NO and O^·−^_2_ (Radi, 2004; Kalyanaraman, 2013). Since ^·^NO in biological systems is in relatively high concentrations, under pathological conditions it may compete with SOD for O^**·**−^_2_. The reaction of ^·^NO and O^·−^_2_ to produce ONOO^−^ is fast (*k* = 6.7 × 10^9^ mol^−1^ s^−1^) (Bartesaghi et al., 2006, 2008, 2010). Despite being a pro-oxidant species, ONOO^−^ reacts slowly and selectively in biological systems. It mainly reacts with protein tyrosine residues to produce 3-nitrotyrosines (Beckman and Koppenol, 1996; Radi, 2004). Protein nitration can be damaging by causing post-translational modifications with pathological outcomes (Webster et al., 2008). Not surprisingly, increased 3-nitrotyrosine residues have been detected in virtually every vascular diseases including PE.

Additionally, ONOO^−^ can also cause DNA damage and lipid structure alteration (Radi, 2004; Webster et al., 2008; Bartesaghi et al., 2010). In PE, 3-nitrotyrosine residues have been observed in normal and complicated pregnancies, predominantly, in endothelium, surrounding smooth muscle and villous stroma (Webster et al., 2006a). One of the key targets of ONOO^−^ in PE is p38 MAPK (p38 mitogen-activated protein kinase), that has been shown to be significantly nitrated in placentas from preeclamptic women compared to normotensive controls (Webster et al., 2006a,b; Myatt, 2010). Activation of the p38 MAPK pathway play an important role in the release of pro-inflammatory cytokines and the induction of enzymes such as COX-2 which controls connective tissue remodeling in pathological conditions, iNOS expression, induction of VCAM-1 and, other adherent proteins along with other inflammatory related molecules (Zarubin and Han, 2005). Nitration of p38 MAPK in PE pregnancies causes a 65% drop on its specific catalytic activity in comparison with normotensive pregnancies (Webster et al., 2006a). The effect of nitration of p38 MAPK in PE is currently under further investigation (Webster et al., 2006a).

## Placental and maternal antioxidant defenses in PE

Antioxidants act as physiological protective agents to prevent oxidative damage caused by high amounts of ROS (Lappas et al., 2010). As with other oxidative stress metabolites, levels of antioxidant enzymes and compounds, such as catalase, superoxide dismutase (SOD) and vitamin E have been found to be variable in PE studies compared to normal pregnancies (Bilodeau, 2014). For example, in a study performed in India, SOD activity, measured in placental tissue and serum from preeclamptic women was found to be 1.4 times higher than in control samples, while glutathione levels remained unchanged (Das et al., 2012). However, other studies have reported that both SOD (Tortladze et al., 2013; Bilodeau, 2014) and catalase activity are decreased in women with PE (Skoczylas-Pietrzyk et al., 1998). One report, on a relatively large cohort from Spain reported lower SOD and higher catalase activities in blood samples of women with PE. In another study from India, lower plasma levels of glutathione where found in preeclamptic women (Kharb et al., 2000; Tortladze et al., 2013). Interestingly, n-acetylcysteine, a precursor of glutathione, has been reported to improve uteroplacental blood flow in *ex-vivo* models of preeclampsia (Bisseling et al., 2004), but in contrast, oral n-acetylcysteine administration have not shown a significant beneficial results in preventing the development of severe preeclampsia (Roes et al., 2006).

In order to improve the understanding in maternal, fetal and neonatal health, in 1986, the Eunice Kennedy Shriver National Institute of Child Health and Human Development from the National Institutes of Health created the Maternal-Fetal Medicine Units Network (MFUM Network, https://mfmu.bsc.gwu.edu). For over 20 years, the MFUM Network have contributed in the area evidence-based medicine in perinatology by unveiling novel and useful therapies in maternal-fetal medicine, including PE. This network completed an important study aimed to evaluate the effect of antioxidant supplementation for the prevention of PE. The study, called Combined Antioxidants and Preeclampsia Prediction Studies (CAPPS), had the objective of determining whether vitamin C and E could reduce the frequency of pregnancy related hypertension. Results from this study showed that the supplementation with vitamin C and E in a low-risk cohort of women demonstrated no significant differences in maternal or newborn complications related to hypertension during pregnancy (Roberts et al., 2010; Weissgerber et al., 2013). On another clinical trial, studying the effects of omega-3 long-chain polyunsaturated fatty acid supplementation to reduce preterm birth, showed no beneficial effects (Harper et al., 2010). Aligned with this findings, the Cochrane Collaboration initiative have concluded on its latest review on antioxidants for preventing PE that evidence from trials reviewed does not support the use of vitamin C and E during pregnancy for the prevention of preeclampsia and other outcomes (Rumbold et al., 2008; Salles et al., 2012).

On the other hand, supplementation with calcium have been demonstrate to play a crucial role for maintaining the production of ^·^NO and preventing PE (López-Jaramillo, 1996; Chen et al., 2013). The Cochrane Collaboration initiative reviewed data from 14 studies (more than 15,000 women) and concluded that calcium supplementation caused a significant reduction in the risk of PE and that this effect was clearly greater in women with low calcium diets (Hofmeyr et al., 2014). This latter result is particularly important in undeveloped countries in where low calcium diets due to poor nutrition is a reality (López-Jaramillo, 1996; López-Jaramillo et al., 2005, 2008) and calcium supplementation might be a low cost alternative for PE prevention.

## Conclusions

Preeclampsia is a pathology characterized by hypertension and proteinuria at the 20th week of gestation that affects 5–10% of the pregnancies. Risk factors for the development of PE includes obesity, insulin resistance and hyperlipidemia that stimulate inflammatory cytokine release and oxidative stress leading to ED. At the molecular level, poor trophoblast invasion and uteroplacental artery remodeling described in PE, increases reactive oxygen species (ROS), hypoxia and ED. Despite all research efforts performed so far, still the etiology of the disease is not known.

Clearly in PE, oxidative stress plays a key role in the development of endothelial dysfunction. However, PE is a very complicated disease that arouses from placental issues that later affect the mother and baby. As we have evidenced, the role of the production of ROS/RNS in PE is still controversial. Particularly, after decades of study there is still no consensus on whether the ^·^NO or ADMA levels are high or low during disease development. In maternal plasma and serum, ^·^NO levels in some important studies have been found to be higher in preeclamptic than in normotensive women. However, studies performed with cutting edge technologies for ^·^NO detection have shown the opposite. Studies performed in placental cells and tissues and umbilical cord cells and blood, have shown that at the placental/fetal stage, ^·^NO levels are lower in PE. However, more research is needed in order to establish the role of ^·^NO in PE.

Nevertheless, superoxide generation in PE from XO or NADPH oxidase has been very well established. Not surprising, most studies have reported higher levels of 3-nitrotyrosine in tissue and fluids from preeclamptic women. Mitochondrial activity is essential in pregnancy because it sustains the metabolic activity of the placenta. In PE, mitochondrial function is known to be disturbed in hypoxic placentas and isolated trophoblasts. This results were expected as mitochondria is known to be affected by exposure to suboptimal environmental conditions as in PE.

Unfortunately, early delivery and patient careful monitoring is still the only way to prevent the fatal effects of the disease. Oral supplementation with antioxidants like vitamin C and E and even n-acetylcysteine have been proven to be a failure on the prevention of the disease. However, so far, clinical trials have shown that calcium supplementation causes a significant reduction in the risk of PE and that this effect was clearly greater in women with low calcium diets.

### Conflict of interest statement

The authors declare that the research was conducted in the absence of any commercial or financial relationships that could be construed as a potential conflict of interest.
